# Comprehensive Transcriptomic Analysis of Mouse Gonadal Development Involving Sexual Differentiation, Meiosis and Gametogenesis

**DOI:** 10.1186/s12575-019-0108-y

**Published:** 2019-10-15

**Authors:** Jian Wang, Geng G. Tian, Zhuxia Zheng, Bo Li, Qinghe Xing, Ji Wu

**Affiliations:** 1 Renji Hospital, Key Laboratory for the Genetics of Developmental & Neuropsychiatric Disorders (Ministry of Education), Bio-X Institutes, School of Medicine, Shanghai Jiao Tong University, Shanghai, 200032 China; 2 Key Laboratory of Fertility Preservation and Maintenance of Ministry of Education, Ningxia Medical University, Yinchuan, 750004 China; 3 State Key Laboratory of Oncogenes and Related Genes, Shanghai Cancer Institute, Renji Hospital, Shanghai Jiao Tong University School of Medicine, Shanghai, 200032 China; 4 Children’s Hospital & Institutes of Biomedical Sciences, Fudan University, 131 Dong-Chuan Road, Shanghai, 200032 China

**Keywords:** Gonad, RNA-Seq, Sex-biased expressed genes, Time series cluster, WGCNA, Alternative splicing

## Abstract

**Background:**

Mammalian gonadal development is crucial for fertility. Sexual differentiation, meiosis and gametogenesis are critical events in the process of gonadal development. Abnormalities in any of these events may cause infertility. However, owing to the complexity of these developmental events, the underlying molecular mechanisms are not fully understood and require further research.

**Results:**

In this study, we employed RNA sequencing to examine transcriptome profiles of murine female and male gonads at crucial stages of these developmental events. By bioinformatics analysis, we identified a group of candidate genes that may participate in sexual differentiation, including *Erbb3*, *Erbb4*, and *Prkg2*. One hundred and two and 134 candidate genes that may be important for female and male gonadal development, respectively, were screened by analyzing the global gene expression patterns of developing female and male gonads. Weighted gene co-expression network analysis was performed on developing female gonads, and we identified a gene co-expression module related to meiosis. By alternative splicing analysis, we found that cassette-type exon and alternative start sites were the main forms of alternative splicing in developing gonads. A considerable portion of differentially expressed and alternatively spliced genes were involved in meiosis.

**Conclusion:**

Taken together, our findings have enriched the gonadal transcriptome database and provided novel candidate genes and avenues to research the molecular mechanisms of sexual differentiation, meiosis, and gametogenesis.

**Supplementary information:**

**Supplementary information** accompanies this paper at 10.1186/s12575-019-0108-y.

## Introduction

Infertility is estimated to affect as many as 48.5 million couples worldwide [1]. Its incidence may be as high as 15%. Following cancer, cardiovascular and cerebrovascular diseases, infertility has become the third major disorder that seriously affects human health and causes considerable stress to patients and their families.

Gonads are the important reproductive organs for mammals. The process of gonadal development includes critical developmental events, such as sexual differentiation, meiosis and gametogenesis. Abnormalities in any of these events may cause infertility. The molecular regulatory mechanisms of these crucial developmental events have been the focus of many studies in reproductive biology.

Many genes that are important for gonadal development have been discovered. For example, the *Sry*, *Sox9*, *M33*, *Dax1*, *Wt1*, *Rspo1*, *Sf1*, *Dmrt1*, *Atrx*, *Wnt4*, *Amh*, *Fgf9*, *Lhr*, *Dhh*, *Insl3*, *Foxl2* and *Sox3* genes play important roles in sexual determination and differentiation [2–5]. The *Stra8*, *Sycp1*, *Sycp2*, *Sycp3*, *Spo11*, *Rce8*, *Dmc1*, *Dazl*, *Mlh1* and *Msh5* genes regulate meiosis [5–9]. The *Figla*, *Lhx8*, *Nobox*, *Sohlh1*, *Sohlh2*, *Bax*, *Ahr*, *Gdf9*, *Pten*, *Scf*, *Bcl2* and *Rps6* genes have roles in follicular and oocyte development [5, 10]. The *Adamts2*, *Bcl2l2*, *Cadm1*, *Ddx25*, *Piwil1*, *Prm1*, *Prm2*, *Tbpl1*, *Tlp*, *Tnp1*, *Tnp2* and *Ube2b* genes are involved in regulating spermatogenesis [5].

Many previous studies of gonadal development used gene knockout mouse models, and only a small number of genes could be studied at a time. Gene chips were also used to research gonadal development [11–14]. Although higher throughput was obtained by gene chip analysis, this research was typically limited to the more highly expressed known genes. Classical gene chip analysis was unable to research unknown or low abundance gene transcripts, nor alternative splicing.

In recent years, high-throughput RNA sequencing (RNA-Seq) overcame the above technical shortcomings of gene chips, and also allowed high-throughput investigation of the whole transcriptome [15]. Previous studies have used RNA-Seq to investigate the development of mouse gonads. However, these studies either focused on single sex gonads at certain developmental stages, or specific cell types in the gonads. Gong et al. investigated the transcriptome profiling of mouse testes at three postnatal ages: 6 days postnatal, 4 weeks old and 10 weeks old, representing infant, juvenile and adult stages, respectively [16]. Pan et al. performed a comparative transcriptomic analysis using RNA-Seq of ovaries isolated from mice aged 1 week and 8 weeks old [17]. McClelland et al. carried out transcriptomic analysis of mouse fetal Leydig cells [18]. Sexual differentiation, meiosis and gametogenesis require the joint participation and interaction of germ and somatic cells in the gonads. Therefore, a better understanding of the mechanisms of sexual differentiation may be obtained by comparing female and male gonadal transcriptomes. It is possible that more insight into the molecular mechanisms of sexual differentiation, meiosis and gametogenesis may be obtained by transcriptomic analysis of murine female and male gonads covering the key stages of developmental corresponding to these critical developmental events. However, such a comparison has not been reported to date.

Here, we systematically compared the transcriptomes of female and male gonads covering the developmental stages corresponding to sexual differentiation, meiosis and gametogenesis. Our study used gonads at 12.5 days post-coitum (dpc), at which time female and male gonads begin to show discernible differences, 13.5 dpc when female germ cells begin to undergo meiosis, and male germ cells begin to stop mitosis, 16.5 dpc when most female germ cells are undergoing meiosis, and male germ cells arrest at the G1/G0 stage, and 6 days post-partum (dpp) when most male germ cells are at the spermatogonial stem cell stage. The current results provide novel candidate genes and potential pathways for further investigation into the molecular mechanisms of sexual differentiation, meiosis and gametogenesis in gonadal development.

## Materials and Methods

### Animals

Eight-week-old C57BL/6 mice were purchased from the SLAC Laboratory Animal Co., Shanghai, China. Mice were housed in a 12 h light: 12 h dark cycle. Protocols and use of animals for this study were approved by the Institutional Animal Care and Use Committee of Shanghai (SYXK-2018-0028) and were conducted in accordance with the National Research Council Guide for Care and Use of Laboratory Animals.

### Gonad Collection and RNA Extraction

Timed matings were performed by placing a male mouse with two females in a cage. Females were checked for the presence of vaginal plugs the next morning. Noon of the day when the mating plug was observed was designated 0.5 dpc. On the relevant days of gestation (12.5, 13.5 and 16.5 dpc), pregnant females were euthanized with carbon dioxide. Embryonic gonads were dissected free of the mesonephros, snap frozen in liquid nitrogen, and stored at − 80 °C. The gonads of 6 dpp mice were also collected, immediately frozen in liquid nitrogen and stored at − 80 °C.

Total RNA was isolated from the gonads of 12.5, 13.5 and 16.5 dpc and 6 dpp mice using TRIzol reagent (Invitrogen, Waltham, Massachusetts, USA) according to the manufacturer’s instructions. An Agilent 2100 Bioanalyzer (Agilent Technologies, Santa Clara, USA) was used to measure total RNA quantity and assess RNA integrity.

### RNA-Seq Library Construction and Sequencing

The cDNA library was constructed with a SMARTer® Ultra Low Input RNA for lllumina® Sequencing kit (Clontech Laboratories, Mountain View, USA). Briefly, first-strand cDNA was synthesized using SMARTScribe Reverse Transcriptase and then purified using SPRI Ampure Beads. After amplification using the Polymerase Mix, double-stranded complementary DNA was purified using SPRI Ampure Beads.

High-throughput sequencing was performed by Shanghai Biotechnology Inc. (Shanghai, China) via 100-nt paired-end sequencing on an Illumina HiSeq 2500 system (Illumina, San Diego, USA).

### Reads Mapping and Measurement of Gene Expression

After sequencing, clean reads were obtained by removing reads containing the adaptor sequences, reads with > 5% ambiguous bases, and low-quality reads, then mapped to the mouse genome (version: mm10_GRCm38) using TopHat software (Version 2.1.1). Gene expression level was calculated using the fragments per kilobase per million mapped reads method.

### Analysis of Differentially Expressed Genes

Analysis of Differentially Expressed Genes (DEGs) was performed by the DESeq package in R language (Version 1.36.0). Genes with a fold change (FC) ≥ 2, and false discovery rate (FDR) < 0.05 were assigned as differentially expressed.

The Series-Cluster analysis of expression profiles of DEGs was performed by using the STEM method (http://www.cs.cmu.edu/~jernst/st/). Significant profiles were identified using the Fisher’s exact test and multiple comparisons.

### Gene Ontology and Kyoto Encyclopedia of Genes and Genomes

Genes were submitted to the databases of Gene Ontology (GO) and Kyoto Encyclopedia of Genes and Genomes (KEGG) for enrichment analysis of the significant GO terms and KEGG pathways. Statistically over-represented GO terms and KEGG pathway categories were obtained by applying a Fisher’s exact *p*-value cutoff < 0.05 and correcting for multiple testing with the Benjamini-Hochberg false discovery rate. Moreover, we built a pathway-act-network of the enriched pathways according to the relationships identified between the pathways in the KEGG database.

### Weighted Gene co-Expression Network Analysis

Weighted gene co-expression network analysis(WGCNA) was used to create a co-expression network using the R package (Version 1.68) according to our previous publication [19]. Briefly, to ensure a scale free topology of the network, an adjacency matrix was established by transforming a pairwise Pearson’s correlation matrix of expression values using a power function. The topological overlap measure (TOM) was calculated using the adjacency matrix, and used to cluster genes with a highly similar co-expression relationship into a network module.

### Alternative Splicing

Alternatively spliced transcripts were identified by the software AS detector (ASD) (Version 1.0) using the Fisher’s exact test as described [20]. Alternative splicing events were adjusted using Jensen-Shannon divergence (FDR < 0.05).

### Quantitative Real-Time Reverse Transcription PCR

Reverse transcription was performed using HiScript II Q RT SuperMix for qPCR (+gDNA wiper) kit (Vazyme, Nanjing, China). The quantitative real-time reverse transcription PCR (qPCR) was performed using FastStart Universal SYBR Green Master Mix (Roche) according to the procedure described previously [21]. The 2^−ΔΔCt^ method was used to calculate the relative expression of genes using the ABI 7500 System Software (V2.0.4), and gene expression levels were normalized to the *Gapdh* level. The primers used for qPCR are shown in Table 1.

**Table 1:** List of the qPCR primers used in this study

Gene Symbol	Primer Sequence
*Sox9*	F: 5′- AGTACCCGCATCTGCACAAC − 3′
	R: 5′- ACGAAGGGTCTCTTCTCGCT − 3′
*Foxl2*	F: 5′- ACAACACCGGAGAAACCAGAC − 3′
	R: 5′- CGTAGAACGGGAACTTGGCTA − 3′
*Sox8*	F: 5′- CGAGGGGATACTGCTGAGG − 3′
	R: 5′- AGCTCTGCGTTATGGAGATGC − 3′
*Wnt4*	F: 5′- AGACGTGCGAGAAACTCAAAG − 3′
	R: 5′- GGAACTGGTATTGGCACTCCT − 3’
*Lgr6*	F: 5′-GAGGACGGCATCATGCTGTC-3’
	R: 5′-GCTCCGTGAGGTTGTTCATACT-3’
*Gas6*	F: 5′-TGCTGGCTTCCGAGTCTTC-3’
	R: 5′-CGGGGTCGTTCTCGAACAC-3’
*Zbtb7c*	F: 5′-TTGATGAGCTGATCGGCATCC-3’
	R: 5′-GTGTTCGGTACTCTTGCTCCT-3’
*Erbb3*	F: 5′-AAGTGACAGGCTATGTACTGGT-3’
	R: 5′-GCTGGAGTTGGTATTGTAGTTCA-3’
*Erbb4*	F: 5′-GTGCTATGGACCCTACGTTAGT-3’
	R: 5′-TCATTGAAGTTCATGCAGGCAA-3’
*Cst9*	F: 5′-GTCCACTGAGAAAGAAAGCTCTG-3’
	R: 5′-CCACTGTGGGAATGAAATGAACA-3’
*Zfp819*	F: 5′-GGGCCGTGTGAGAGATTGG-3’
	R: 5′-GCCTGGAATACCCCACTACC-3’
*Rec8*	F: 5′-TATGTGCTGGTAAGAGTGCAAC-3’
	R: 5′-TGTCTTCCACAAGGTACTGGC-3’
*Gapdh*	F: 5′- CATGGCCTTCCGTGTTCCTA − 3’
	R: 5′- GCCTGCTTCACCACCTTCTT −3’

### Statistical Analysis

For each group, three independent experiments were replicated. The data are expressed as the mean ± SEM. Data were statistically analyzed with ANOVA, followed by the Fisher’s least significant difference test with R software (Version 3.5.1). Differences were considered significant at *p* < 0.05.

## Results

### Sex-Biased Gene Expression in Mouse Gonads

In order to screen candidate genes regulating gonadal sexual differentiation, we initially screened the differentially expressed genes in female and male gonads at the same stage of development. Wayne analysis was then performed to identify the genes with higher expression in all four stages of development, and these genes were defined as sex-biased expressed genes that may participate in regulating gonadal sexual differentiation.

**Fig. 1:**
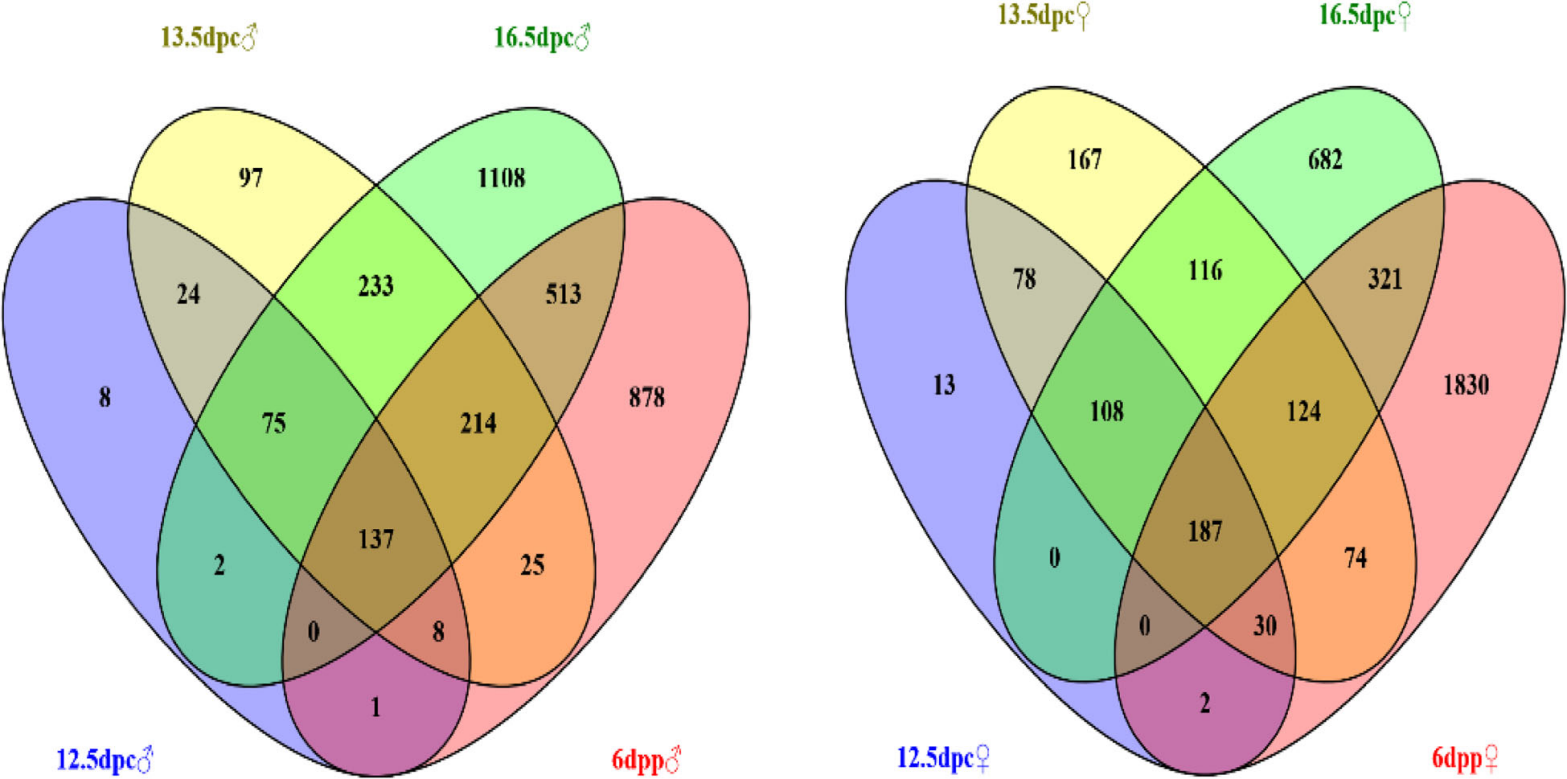
Venn diagram of genes with higher expression in murine male (left) and female (right) gonads at different stages of development

One hundred and thirty-seven male-biased and 187 female-biased expressed genes were obtained (Fig. 1 and Additional file 3 Table. S1, Additional file 4 S2). These genes were classified into 3 categories. The first category included genes functionally annotated and reported to play an important role in sex determination and differentiation, such as *Sox9* [22, 23], *Xist* [24], *Amh* [25], *Insl3* [26], *Emx2* [26], *Dhh* [26], *Wnt4* [27, 28], *Foxl2* [29] and *Fst* [26]. The second category included genes with functional annotations but no reported involvement in sex determination and differentiation, including *Erbb3*, *Mt3*, *Gas6*, *Lzts1*, *Lrrn1*, *Olfm1* and *Lgr6*. The third category had genes with no functional annotations, such as *Gm30587*, *GM6705*, *Gm11734* and *Gm31399*.

### Validation of Representative Sex-Biased Gene Expression by qPCR

**Fig. 2:**
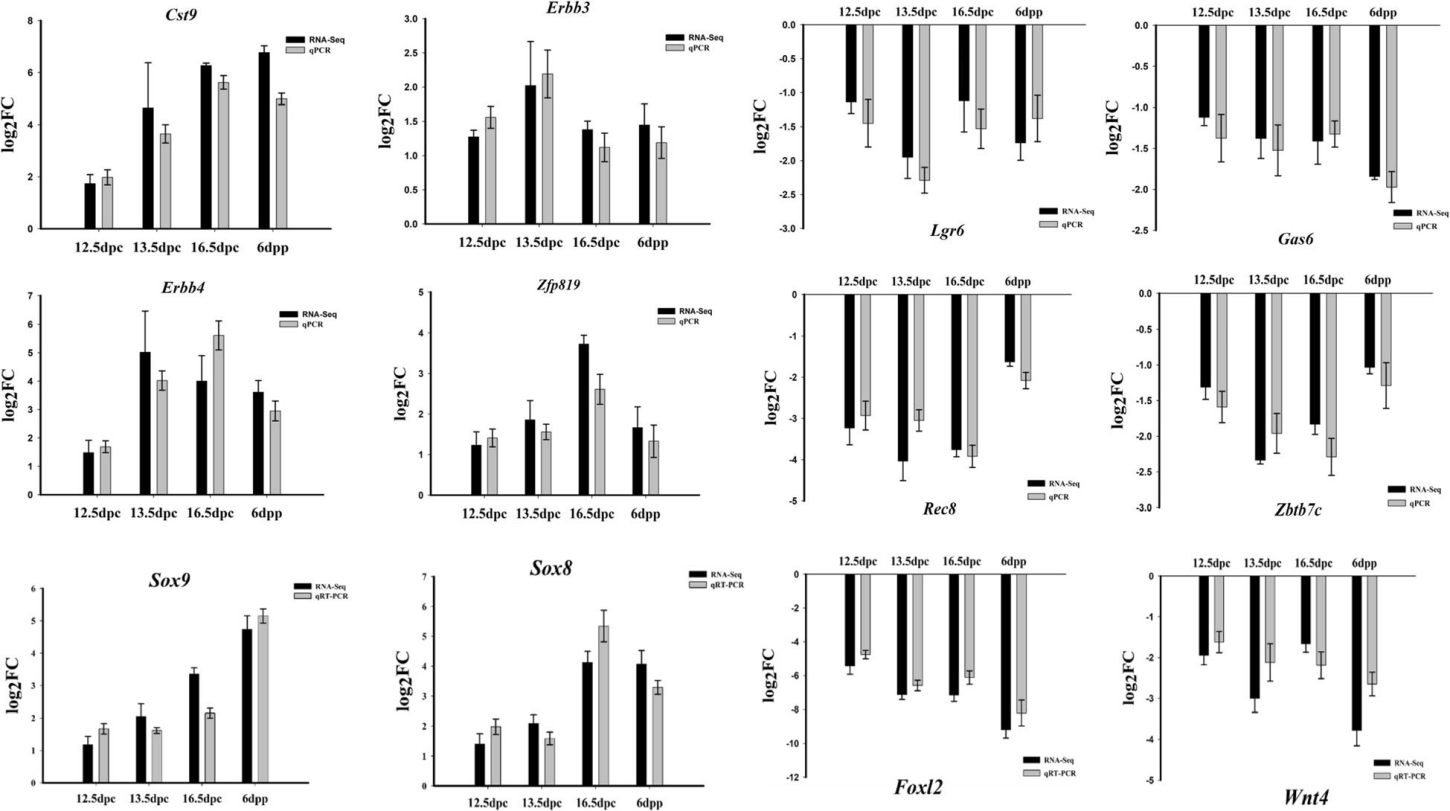
Validation of the identified sex-biased gene expression by qPCR. log2FC: Male/Female

To validate the screened sex-biased expressed genes, twelve genes (*Cst9, Zbtb7c, Lgr6*, *Gas6*, *Erbb3*, *Erbb4, Zfp819, Rec8*, *Sox8*, *Sox9*, *Wnt4* and *Foxl2*) were randomly selected and their ovarian and testicular expression levels at different stages of development were detected by qPCR. The results showed that *Cst9, Erbb3*, *Erbb4, Zfp819, Sox8* and *Sox9* had biased expression in male gonads, whereas *Lgr6*, *Zbtb7c*, *Gas6*, *Rec8*, *Wnt4* and *Foxl2* had biased expression in female gonads (Fig. 2). The results of qPCR were consistent with RNA-seq.

### Protein–Protein Interaction Network of Mouse Gonadal Sex-Biased Gene Expression

The protein–protein interaction (PPI) network of mouse gonadal sex-biased expressed genes was constructed by using String and Cytoscape software. Twenty nodal genes were connected via more than 10 edges in the network; *Wnt4*, *Dhh*, *Amh*, *Bmp2*, *Fst*, *Sox9*, *Spp1*, *Cd44*, *Cyp11a1*, *Cyp17a1*, *Erbb3*, *Erbb4*, *Fshr*, *Lhcgr*, *Mapk4*, *Prkg2*, *Calb1*, *Nos1*, *Acta2* and *Cacna1d* (Additional file 1 Figure S1 and Tab. 2). There were defined as hub genes in the network, suggesting they may play a key regulatory role in the process of gonadal sexual differentiation.

**Table 2:** Hubs of the protein–protein interaction network constructed from the mouse gonadal sex-biased expressed genes

Gene Symbol	Definition	Gene type
*Wnt4*	wingless-type MMTV integration site family, member 4	protein coding
*Dhh*	desert hedgehog	protein coding
*Amh*	anti-Mullerian hormone	protein coding
*Bmp2*	bone morphogenetic protein 2	protein coding
*Fst*	follistatin	protein coding
*Sox9*	SRY (sex determining region Y)-box 9	protein coding
*Spp1*	secreted phosphoprotein 1	protein coding
*Cd44*	CD44 antigen	protein coding
*Cyp11a1*	cytochrome P450, family 11, subfamily a, polypeptide 1	protein coding
*Cyp17a1*	cytochrome P450, family 17, subfamily a, polypeptide 1	protein coding
*Erbb3*	erb-b2 receptor tyrosine kinase 3	protein coding
*Erbb4*	erb-b2 receptor tyrosine kinase 4	protein coding
*Fshr*	follicle stimulating hormone receptor	protein coding
*Lhcgr*	luteinizing hormone/choriogonadotropin receptor	protein coding
*Mapk4*	mitogen-activated protein kinase 4	protein coding
*Prkg2*	protein kinase, cGMP-dependent, type II	protein coding
*Calb1*	calbindin 1	protein coding
*Nos1*	nitric oxide synthase 1, neuronal	protein coding
*Acta2*	actin, alpha 2, smooth muscle, aorta	protein coding
*Cacna1d*	calcium channel, voltage-dependent, L type, alpha 1D subunit	protein coding

Among these 20 hub genes, *Sox9*, *Cyp17a1*, *Cyp11a1*, *Dhh*, *Amh*, *Fshr*, *Lhcgr*, *Wnt4*, *Fst* and *Bmp2* are well-known sex differentiation genes. The function of the other 10 hub genes with respect to sexual differentiation remains unknown. Interestingly, *Erbb3*, *Erbb4*, *Prkg2* and *Mapk4* encode protein kinases. Protein kinases catalyze protein phosphorylation and transfer the γ-phosphate groups of adenosine triphosphate (usually ATP) to amino acid residues of substrate proteins. The four protein kinase genes suggest that protein phosphorylation may play an important role in sexual differentiation.

### Time Series Cluster Analysis of Gonadal Gene Expression during Development

**Fig. 3:**
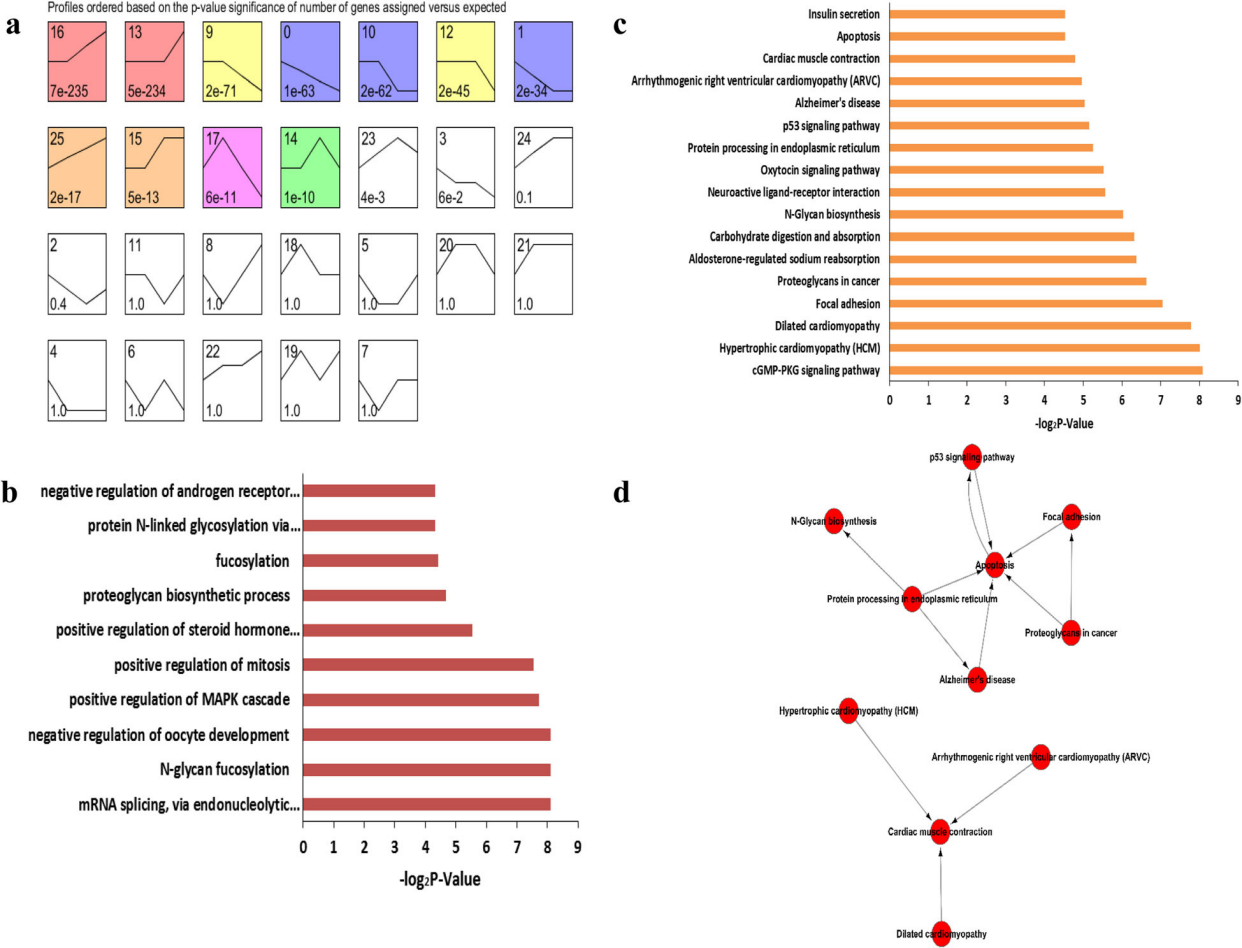
Time series cluster analysis of gene expression during female gonadal development. **a** Differentially expressed genes in the developing female gonads were separated into 26 possible model profiles, including 11 with significant changes in gene expression. **b** GO enrichment analysis of genes in profile 25 from the female gonad. **c** KEGG enrichment analysis of genes in profile 25 from the female gonad. **d** Pathway-Act-Network analysis

**Fig. 4:**
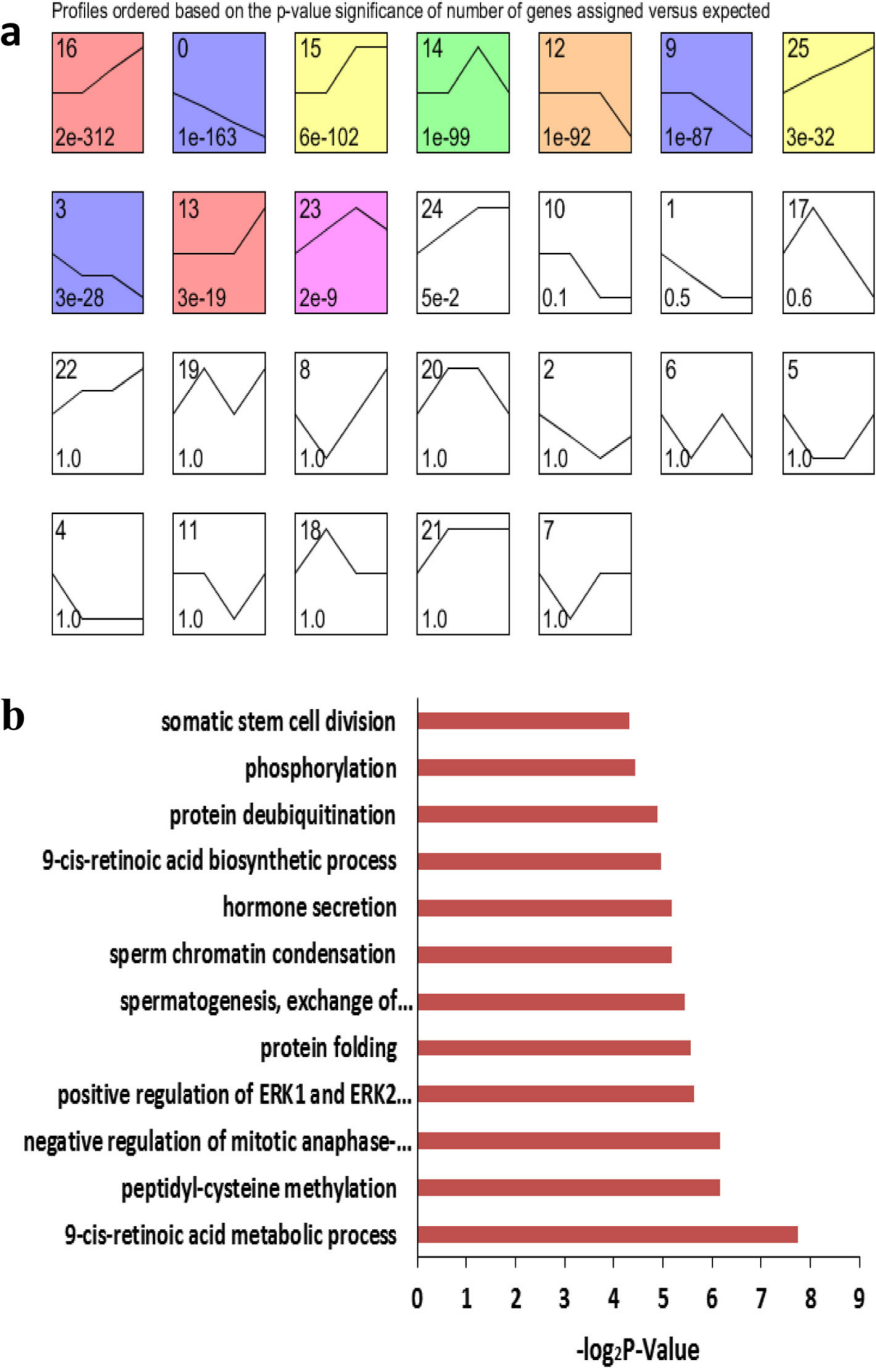
Time series cluster analysis of gene expression during male gonadal development. **a** Differentially expressed genes in the developing male gonads were separated into 26 possible model profiles, including 10 with significant changes in gene expression. **b** GO enrichment analysis of genes in profile 25 from the male gonad

Time series cluster analysis was performed to identify the global trends and model profiles of gonadal gene expression according to signal densities in the 12.5, 13.5 and 16.5 dpc, and 6 dpp temporal sequence. We identified 26 possible gene expression profile patterns (numbered 0–25), which represent the overall expression patterns (Fig. 3a, 4a). Eleven patterns (No. 0, 1, 9, 10, 12, 13, 14, 15, 16, 17 and 25) showed significance (*P* < 0.05) in female gonadal development, and 10 patterns (No. 0, 3, 9, 12, 13, 14, 15, 16, 23 and 25) showed significance (P < 0.05) in male gonadal development (Fig. 3a, 4a).

For both female and male gonads, genes in pattern No. 25 showed increased expression levels during gonadal development, suggesting these genes may be crucial for gonadal development. Significantly enriched GO terms from pattern No. 25 are illustrated in Fig. 3b and 4b. For the female gonads, pattern No. 25 contained 102 genes and significantly enriched GO terms closely correlated with mRNA splicing, positive regulation of the MAPK cascade, N-glycan fucosylation, and negative regulation of oocyte development (Fig. 3b). This list was also significantly enriched for genes associated with the p53 signaling, N-Glycan biosynthesis signaling, and cGMP-PKG signaling pathways (Fig. 3c). To further understand the importance of pathway interactions, and to screen key pathways for significant roles in female gonadal development, we built a Pathway-Act-Network according to the direct or systemic interactions assigned between pathways in the KEGG database (Fig. 3d). Key pathways were identified, including apoptosis signaling and protein processing in endoplasmic reticulum signaling pathways, as shown in Fig. 3d. In addition, we found 9 unannotated ncRNAs in pattern No. 25, designated *LOC102639808*, *LOC102640507*, *LOC102637464*, *LOC102637824*, *LOC102638234*, *LOC102634191*, *LOC102635948*, *LOC102637233* and *LOC102631989*.

Similarly, for the male gonads, pattern No. 25 contained 134 genes and had significantly enriched GO terms closely correlated with phosphorylation, protein deubiquitination, 9-cis-retinoic acid biosynthesis and sperm chromatin condensation (Fig. 4b). This list also included genes that were significantly enriched in mucin type O-glycan biosynthesis signaling and calcium signaling pathways. Seventeen unannotated ncRNAs were found in this pattern, designated *LOC102631654*, *LOC102631845*, *LOC102632148*, *LOC102632349*, *LOC102634896*, *LOC102635066*, *LOC102635096*, *LOC102636262*, *LOC102636409*, *LOC102637414*, *LOC102637640*, *LOC102637873*, *LOC102637942*, *LOC102639461*, *LOC102640347*, *LOC102640483* and *LOC102640949*.

### Weighted Gene Co-Expression Network Analysis

**Fig. 5:**
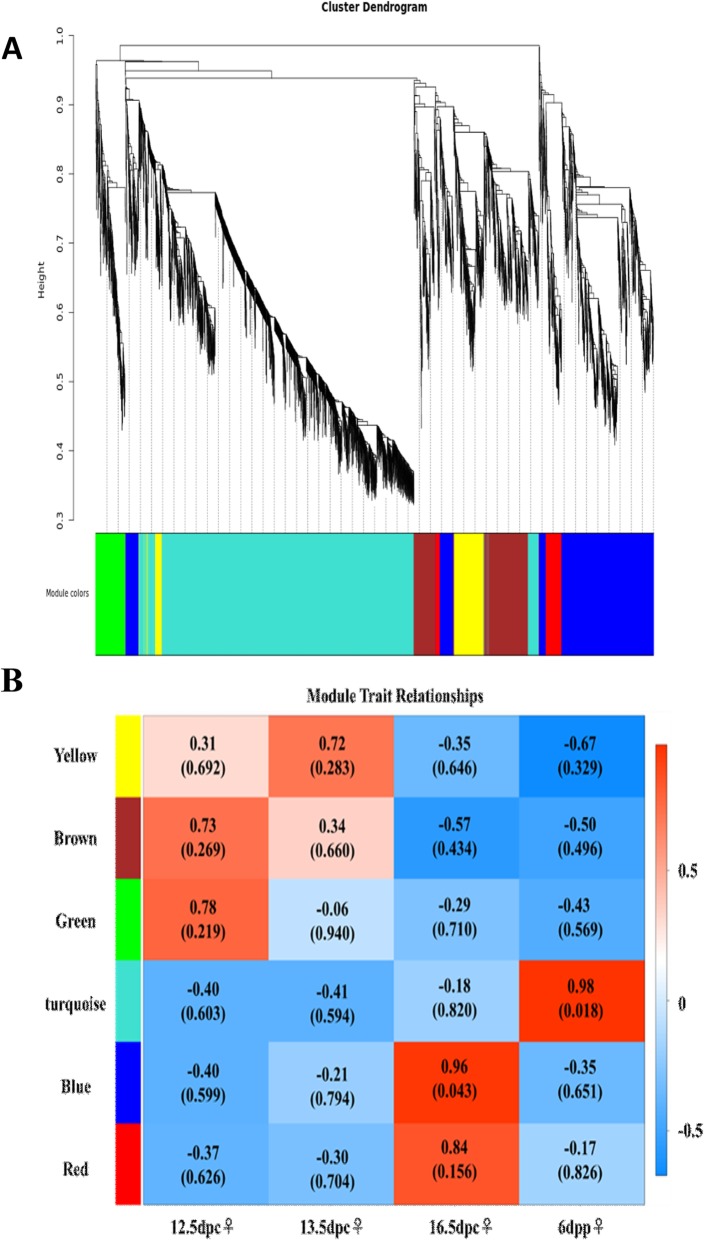
Weighted gene co-expression network analysis **(**WGCNA). **a** Hierarchical cluster tree shows the six co-expression modules identified using WGCNA. **b** Heatmap reporting the correlation between modules. **c** Gene co-expression network of meiosis-related genes in the blue module

In mouse female gonads, some germ cells were reported to enter meiosis at 13.5 dpc [7, 30], and most germ cells were undergoing meiosis at 16.5 dpc [6, 7]. To study genes that regulate meiosis in germ cells, transcriptome profiles from female gonads were subjected to WGCNA. Using this unsupervised and unbiased analysis, we identified six distinct co-expression modules corresponding to clusters of correlated transcripts (Fig. 5). The co-expression relationship of modules between samples is shown in Fig. 5b. The GO functional enrichment analysis was performed for genes in each module. Genes in the blue module were found to be significantly enriched for meiotic GO items, suggesting that genes in the blue module may be closely associated with meiosis.

In order to obtain core genes that regulate meiosis during gonadal development from 12.5 dpc to 6 dpp, genes significantly enriched for meiosis according to GO analysis of the blue module were selected to construct a gene co-expression network (Additional file 2 Fig. S2). According to co-expression relationships, *Mei1*, *Meiob* and *Sycp2* were the core genes in the network, suggesting they may have a crucial role in the regulation of meiosis during gonadal development from 12.5 dpc to 6 dpp.

### Alternative Splicing

**Fig. 6:**
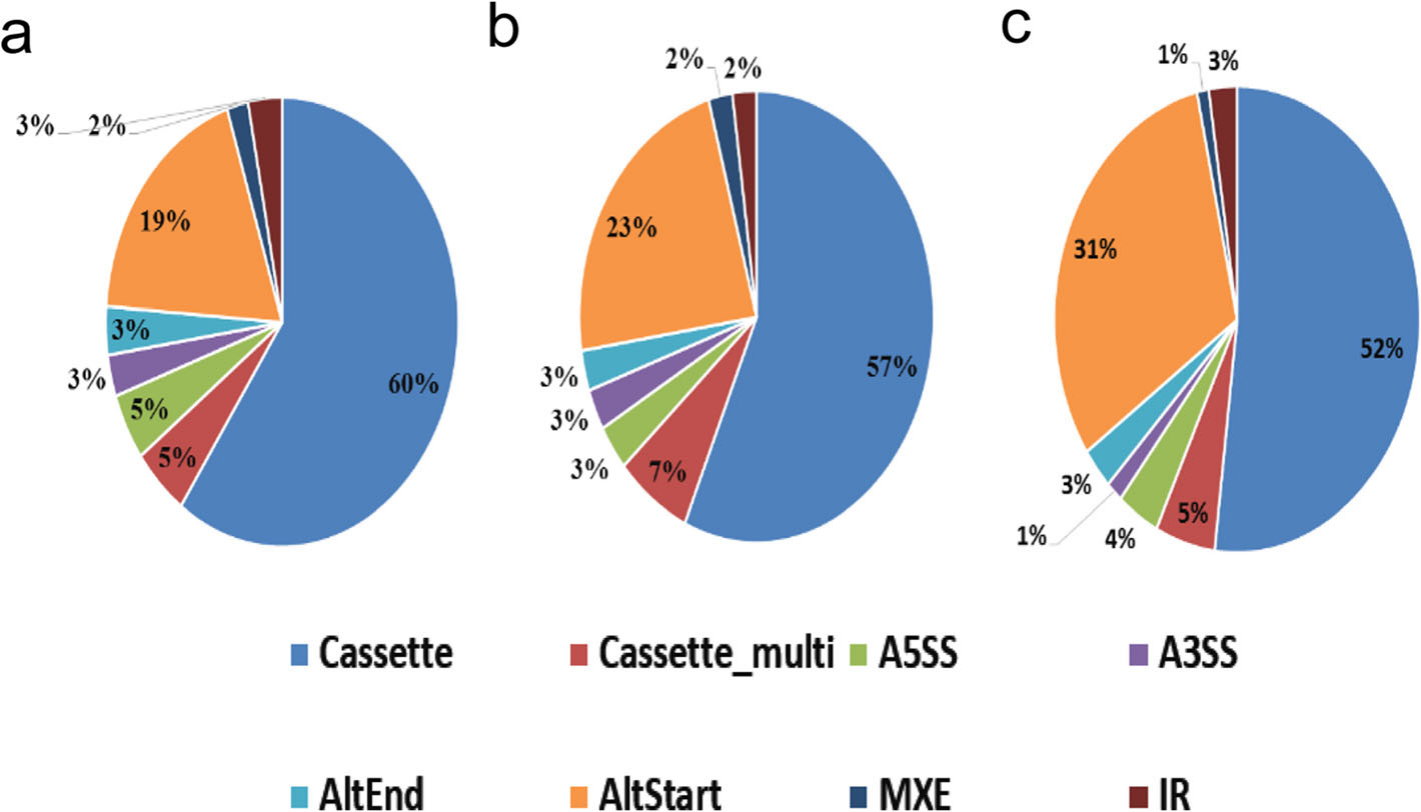
Composition of eight types of alternative splicing (AS) patterns in mouse gonads at different stages of developmental stages and during sexual differentiation. **a** Composition of eight AS patterns in female mouse gonads at different developmental stages. **b** Composition of eight AS patterns in male mouse gonads at different developmental stages. **c** Composition of eight AS patterns in gonads during sexual differentiation

Alternative splicing is regulated during gene expression and results in a single gene encoding multiple mRNAs and proteins. In this process, particular exons of a gene may be included or excluded from the final processed mRNA. Consequently, the proteins translated from alternatively spliced mRNAs may differ in their amino acid sequences and biological functions. Alternative splicing events of transcripts during mouse gonadal development and sexual differentiation were predicted by software AS detector; 1902 and 1559alternative splicing events were identified during female and male gonadal development, respectively, and 962 alternative splicing events were identified during gonadal sexual differentiation. Cassette exons and alternate start sites were the main forms of alternative splicing during murine gonadal development and sexual differentiation (Fig. 6).

It is noteworthy that GO functional enrichment analysis of the differentially-expressed and alternatively spliced genes found that a considerable number were involved in meiosis (Additional file 5 Tab. S3). For example, *Dazl* was found to exhibit cassette-type alternative splicing at exon 9. *Dmc1* was shown to have cassette-type alternative splicing at exons 6, 8 and 11, and multiple cassette-type alternative splicing at exons 7 and 9. These results suggest that alternative splicing may be involved in regulating meiosis.

### Dynamic Expression Patterns of miRNAs Involved in Gametogenesis

**Fig. 7:**
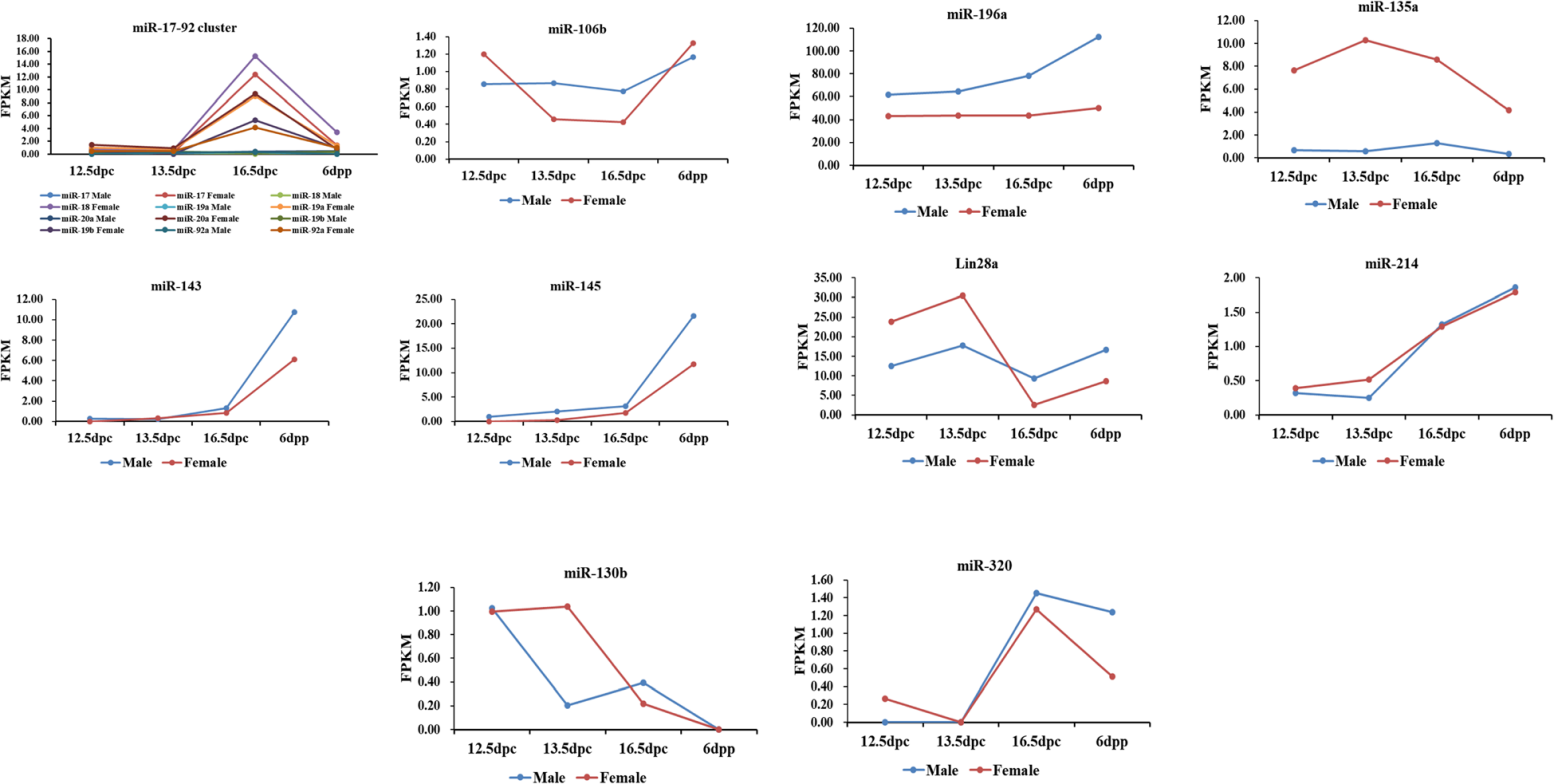
Expression patterns of miRNAs involved in gametogenesis

Distinct miRNAs contribute to gametogenesis [31, 32]. The abnormal expression of specific miRNAs is associated with certain human reproductive dysfunctions. The expression level of such miRNAs can be used as molecular biomarkers to diagnose infertility [33]. In order to study the dynamic expression pattern of miRNAs involved in gametogenesis, we entered “miRNA”, “oogenesis”, “spermatogenesis”, “ovary”, and “testis” keywords into PubMed literature searches. Some miRNAs were previously reported to regulate gametogenesis [34, 35]. We then analyzed the expression of these miRNAs in the gonads of both sexes at embryonic stage (12.5, 13.5 and 16.5 dpc) and infant stage (6 dpp), as shown in Fig. 7.

Most of the miRNAs were expressed in both embryonic and infant stage gonads. The dynamic expression patterns of miR-143, miR-145, miR-214, Lin28 and miR-320 were consistent in the gonads of both sexes. Among them, the expression levels of mir-143, mir-145 and mir-214 generally increased with gonadal development. The expression patterns of miR-130b, miR-17-92, miR-106b, miR-196a and miR-135a showed sexual dimorphism, and mir-135a exhibited female-biased expression.

## Discussion

Although the testes and ovaries have different structures and functions, they develop from a common sexually undifferentiated bipotential anlage [36]. Sexual differentiation is essential for gonadal development and function. However, owing to the complexity of the event, the underlying molecular mechanisms remain to be fully understood and require further research. We screened 137 male-biased and 187 female-biased expressed genes, which may be important candidates for regulating gonadal sexual differentiation.

Some classic genes known to play an important role in sexual differentiation were found in the identified genes of this study, such as *Sox9*, *Xist*, *Foxl2*, *Wnt4*, *Amh*, *Insl3*, *Dhh*, *Emx2* and *Fst*, indicating that our method for screening target genes was reliable. For instance, *Sox9* is known to be highly expressed in pre-Sertoli cells of male gonads, and plays a key role in male sex determination by the upregulation of genes associated with testicular differentiation (e.g. *Amh*, *Fgf9*, *Dhh* and *Pdgf*) and downregulation of genes associated with ovarian development (e.g. *Wnt4* and *Ctnnb1*) [37]. Homozygous loss of *Sox9* in XY mice leads to ovarian development and *Sox9* overexpression in XX mice leads to testicular development [23]. Mishina et al. reported that Sertoli cells of the fetal testis produce anti-Müllerian hormone (AMH) that causes the regression of the Müllerian ducts, the anlagen of female reproductive organs (uterus, oviducts, and upper portion of the vagina) [38]. The expression of *Wnt4* is required for female sexual differentiation. In XX individuals, activation of the β-catenin signaling pathway by secreted RSPO1 and WNT4 proteins is required for granulosa cell differentiation and functional ovarian development [27].

The sex-biased gene expression that we identified included some transcripts without functional annotation with regard to sexual differentiation, as well as transcripts lacking any functional annotation. The current study has provided a large number of candidate genes and the opportunity to identify novel transcripts regulating gonadal sexual development. For example, *Lgr6* exhibited female-biased expression, suggesting it may play a role in female sexual differentiation. *Lgr6* encodes a receptor for R-spondins (RSPO1, RSPO2, RSPO3 or RSPO4), and it associates with phosphorylated LRP6 and frizzled receptors that are activated by extracellular Wnt receptors, triggering the canonical Wnt signaling pathway to increase expression of target genes. Kawasaki et al. reported that *Lgr6* is involved in tooth development [39], but it remains unknown if *Lgr6* plays a role in sexual differentiation.

We identified a group of genes with expression levels that continued to rise during female or male gonadal development, suggesting that these genes may be crucial for gonadal development. A significant proportion of these genes were ncRNAs that have not yet been functionally annotated, such as *LOC102639808*, *LOC102640949*, *LOC102640507*, and *LOC102637464*. This provides a valuable opportunity to identify previously unknown genes that regulate gonadal development. Our future work will investigate the function of these ncRNAs in the regulation of gonadal development. In addition, candidate genes that may be crucial for the development of female gonads were subjected to GO and KEGG pathway enrichment analysis. We identified GO terms and signaling pathways associated with protein glycosylation, suggesting this modification may be involved in the regulation of female gonadal development. Our future studies will target the role of protein glycosylation in the development of female gonads.

Meiosis is the unique division of reproductive cells that produces haploid gametes. The molecular mechanisms regulating meiosis are very complex and have not been fully elucidated. In the gonads of female mice, germ cells were reported to first enter the meiotic cell cycle at 13.5 dpc [7, 30], with most germ cells undergoing meiosis at 16.5 dpc [6, 7]. Transcriptome profiles of female gonads provide valuable data sources for studying the molecular mechanisms of meiosis. Therefore, WGCNA was used to analyze the female gonadal transcriptome data, revealing a co-expression module of genes related to meiosis. A co-expression network was constructed and three hub genes *(Mei1*, *Meiob* and *Sycp2*) were identified that may play a core regulatory role in meiosis during gonadal development from 13.5 dpc to 6 dpp. Reinholdt et al. reported that *Mei1* was required for vertebrate meiosis, and was positioned upstream of *Dmc1* in the genetic pathway that operated during mammalian meiosis [40]. Luo et al. reported that MEIOB exhibited single-stranded DNA-binding and exonuclease activities and was essential for meiotic recombination in both sexes [41]. MEIOB was found to be a meiosis-specific paralogue of RPA1, and co-localized with RPA in foci on meiotic chromosomes. MEIOB formed a complex with RPA and SPATA22. The chromatin localization and stability of MEIOB depended on SPATA22, and vice versa [41]. *Meiob*-null mice exhibited meiotic failure and sterility of both sexes [41]. During meiosis, the arrangement of homologous chromosomes is tightly regulated by the synaptonemal complex [42]. Each synaptonemal complex consists of two axial/lateral elements, and numerous transverse filaments [42]. Yang et al. reported that SYCP2 was a primary determinant of axial/lateral elements and was required for the incorporation of SYCP3 into synaptonemal complexes [42]. The fertility of homozygous *Sycp2* mutant mice was sexually dimorphic; males were sterile because of a block in meiosis, whereas females were subfertile with reduced litter sizes [42].

Alternative splicing events for gene transcripts during murine gonadal development and sexual differentiation were predicted by the software AS detector. Interestingly, a considerable portion of differentially expressed and alternatively spliced genes were associated with meiosis, suggesting that meiosis may be regulated by alternative splicing. Substantial evidence suggests that pre-mRNA splicing is an important regulator of mouse spermatogenesis [43]. The stage-enriched expression of splicing proteins and substantial changes in alternative splicing patterns around meiosis suggest that alternative splicing may be critical for the mitotic-to-meiotic transition during mouse spermatogenesis [43]. Liu et al. reported that male mice were sterile after *Bcas2*, an alternative splicing regulator, was knocked out in spermatogonial stem cells [43]. Although the spermatogonia were grossly normal, spermatocytes in meiotic prophase I were scarce and meiotic events were absent in the BCAS2-depleted testes [43]. Moreover, these results suggested that BCAS2 may be involved in the splicing of *Dazl*, an intrinsic germ cell factor that promotes the initiation of meiosis [43]. However, the regulation of alternative splicing and its role in meiosis may be very complex, and the current research may be the tip of the iceberg.

It is known that miRNA plays an important regulatory role in gametogenesis [31, 32]. Polycystic ovary syndrome, premature ovarian failure and other reproductive disorders are associated with the abnormal expression of specific miRNAs. Selected miRNAs, which contain about 22–25 nucleotides, are often used as a molecular diagnostic markers for infertility owing to the advantages of the small molecular size [33]. When screening for sex-biased gene expression, we found that mir-135a showed female-biased expression, suggesting that it may be involved in regulating female sexual differentiation. Moritoki et al. reported that miR-135a contributed to the maintenance of spermatogonial stem cells by regulating FoxO1 [44]. However, there have been no reports on its involvement in sexual differentiation.

We studied the dynamic expression changes of miRNAs associated with gametogenesis in embryonic and early postnatal gonads, and found that the expression level of mir-196a was higher in male compared with female gonads. The expression level of mir-196a showed an upward trend during the development of male gonads, suggesting it may be important for gonadal development. Rah et al. reported that a putative gene-gene interaction between miR-146 and miR-196a2 may be involved in the development of premature ovarian failure [45]. However, whether this interaction is involved in the regulation of male gonadal development remains to be studied. It is noteworthy that during female gonadal development, the expression of all six members of the miR-17-92 cluster (miR-17, miR-18, miR-19a, miR-20a, miR-19b and miR-92a), was significantly higher at 16.5 dpc than that at other developmental periods. This is a critical stage of female germ cell development, suggesting that mir-17-92 may be involved in regulating oogenesis. In agreement with this finding, our past research showed that ablation of the mir-17-92 cluster in germ cells caused subfertility in female mice, and miR-17-92 participated in the regulation of oogenesis [21].

In this study, the murine transcriptomes of female and male gonads were systematically compared during stages of development corresponding to sexual differentiation, meiosis and gametogenesis. We obtained good quality and reliable transcriptome data from gonads during the critical periods of the above developmental stages, enriching the overall mouse gonadal transcriptome data. Some unannotated candidate genes that may participate in the regulation of sexual differentiation and development were also identified. Our current findings provide novel candidate genes and avenues for further research into the molecular mechanisms of sexual differentiation, meiosis, gametogenesis and other key developmental events in gonadal development.

## Conclusions

Our findings have enriched the gonadal transcriptome database and provided novel candidate genes and avenues to research the molecular mechanisms of sexual differentiation, meiosis, gametogenesis, and other key developmental events in gonadal development.

## Supplementary information

**Additional file 1: Figure S1.** Protein–protein interaction network for genes with sex-biased gonadal expression in mice. (PDF 203 kb)

**Additional file 2: Figure S2.** Gene co-expression network of meiosis-related genes in the blue module. (PDF 478 kb)

**Additional file 3: Table S1.** The list of male-biased expressed genes. (DOCX 15 kb)

**Additional file 4: Table S2.** The list of female-biased expressed genes. (DOCX 16 kb)

**Additional file 5: Table S3.** A list of differential alternative splicing meiotic genes. (DOC 66 kb)

## Data Availability

Sequence data were submitted to the GEO database under accession number GSE117590.

## Abbreviations

AMH: Anti-Müllerian hormone
ASD: Software AS detector
DEGs: Differentially Expressed Genes
Dpc: Days post-coitum
Dpp: Days post-partum
FDR: False discovery rate
GO: Gene Ontology
KEGG: Kyoto Encyclopedia of Genes and Genomes
PPI: The protein–protein interaction
RNA-Seq: High-throughput RNA sequencing
TOM: The topological overlap measure
WGCNA: Weighted gene co-expression network analysis
