# Supplementary figures and images for: Comprehensive Transcriptomic Analysis of Mouse Gonadal Development Involving Sexual Differentiation, Meiosis and Gametogenesis
Source: Biol Proced Online. 2019 Oct 15;21:20. doi: 10.1186/s12575-019-0108-y (PMC6794783; doi:10.1186/s12575-019-0108-y)

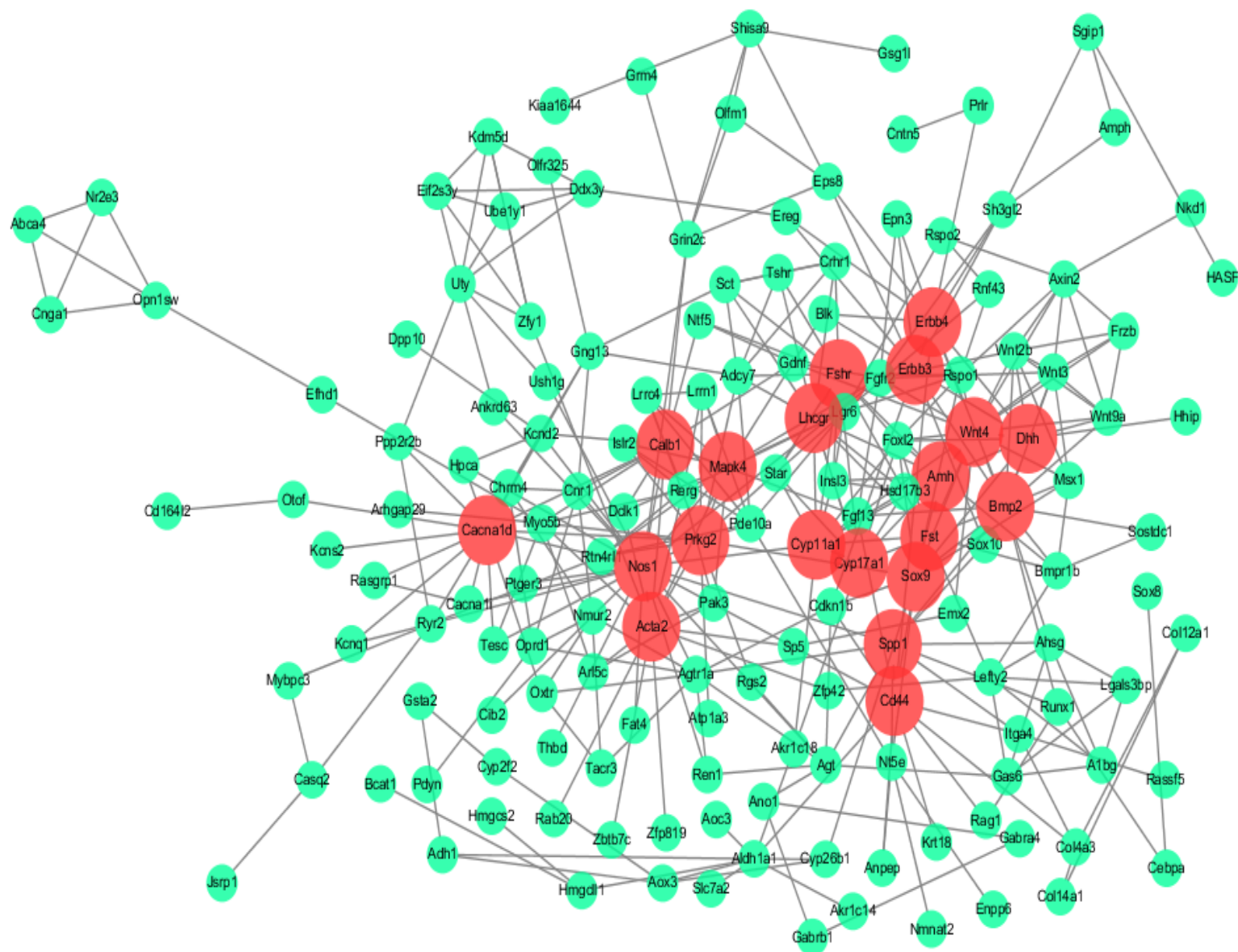

Supplement: Supplementary file 1 — Additional file 1: Figure S1. Protein–protein interaction network for genes with sex-biased gonadal expression in mice. (PDF 203 kb) [file 12575_2019_108_MOESM1_ESM.pdf]

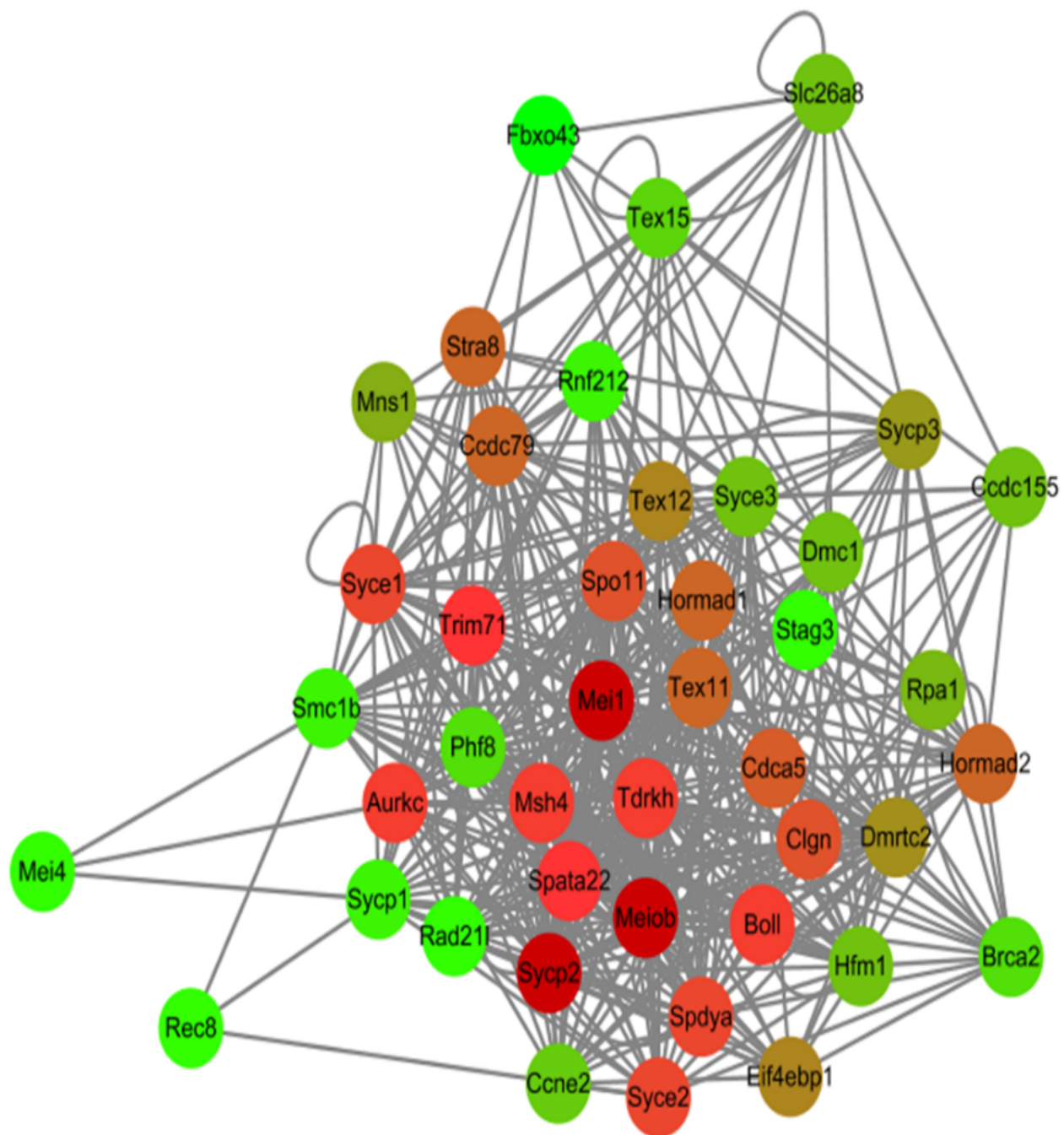

Supplement: Supplementary file 2 — Additional file 2: Figure S2. Gene co-expression network of meiosis-related genes in the blue module. (PDF 478 kb) [file 12575_2019_108_MOESM2_ESM.pdf]
