# Supplementary material for: Comprehensive Transcriptomic Analysis of Mouse Gonadal Development Involving Sexual Differentiation, Meiosis and Gametogenesis
Source: Biol Proced Online. 2019 Oct 15;21:20. doi: 10.1186/s12575-019-0108-y (PMC6794783; doi:10.1186/s12575-019-0108-y)
Supplement: Supplementary file 3 — Additional file 3: Table S1. The list of male-biased expressed genes. (DOCX 15 kb) [file 12575_2019_108_MOESM3_ESM.docx]

**Table S1 The list of male-biased expressed genes**

| **Male Biased** | | | | |
| --- | --- | --- | --- | --- |
| *Aard* | *Col4a3* | *Gm10863* | *Mt3* | *Slc13a2* |
| *Acta2* | *Crhr1* | *Gm7049* | *Mybpc3* | *Slc13a2os* |
| *Adcy7* | *Cst9* | *Gm7329* | *Nkx3-1* | *Slc16a7* |
| *Adh1* | *Cyp11a1* | *Gramd1b* | *Nt5e* | *Slc38a5* |
| *Agt* | *Cyp17a1* | *Grin2c* | *Ntf5* | *Sostdc1* |
| *Ahsg* | *Cyp26b1* | *Gsg1l* | *Olfr329-ps* | *Sowahb* |
| *Aldh1a1* | *Ddx3y* | *Gsta2* | *Otof* | *Sox10* |
| *Amh* | *Defb19* | *Hes5* | *Oxtr* | *Sox8* |
| *Ankrd63* | *Dhh* | *Hhip* | *Padi2* | *Sox9* |
| *Aoc3* | *Dtna* | *Hsd17b3* | *Pak3* | *Spic* |
| *Atp1a3* | *Dtx4* | *Insl3* | *Pdyn* | *Spp1* |
| *BC006965* | *Efhd1* | *Islr2* | *Prlr* | *Star* |
| *Bcan* | *Eif2s3y* | *Itga4* | *Prss35* | *Stc1* |
| *Bend7* | *Enc1* | *Jakmip2* | *Rab20* | *Tacr3* |
| *Blk* | *Enpp6* | *Jsrp1* | *Rasgef1c* | *Tesc* |
| *C7* | *Eps8* | *Kazald1* | *Rassf5* | *Thbd* |
| *Cadps* | *Erbb3* | *Kcns2* | *Ren1* | *Tmem184a* |
| *Casq2* | *Erbb4* | *Kdm5d* | *Rerg* | *Tmem200b* |
| *Cbln1* | *Etd* | *Lefty2* | *Rimbp2* | *Trim47* |
| *Cbln4* | *Fam19a2* | *Lhcgr* | *Rims4* | *A330041J22Rik* |
| *Cebpa* | *Fgf13* | *Mapk4* | *Rnf207* | *A730049H05Rik* |
| *Cecr6* | *Frzb* | *Me1* | *Rtn4rl1* | *LOC101055983* |
| *Cerkl* | *Fshr* | *Mgarp* | *Sct* | *LOC102632536* |
| *Cited1* | *Fstl4* | *Mmd2* | *Sel1l3* | *LOC102635699* |
| *Cnga1* | *Gdnf* | *Mro* | *Sgip1* | *LOC102635707* |
| *Col14a1* | *Wnt3* | *Msc* | *Sh3gl2* | *4930486L24Rik* |
| *Uty* | *Ush1g* | *Zfp819* | *Zfy1* | *E230013L22Rik* |
| *Uba1y* | *Tspan11* |  |  |  |
