# Supplementary material for: Comprehensive Transcriptomic Analysis of Mouse Gonadal Development Involving Sexual Differentiation, Meiosis and Gametogenesis
Source: Biol Proced Online. 2019 Oct 15;21:20. doi: 10.1186/s12575-019-0108-y (PMC6794783; doi:10.1186/s12575-019-0108-y)
Supplement: Supplementary file 4 — Additional file 4: Table S2. The list of female-biased expressed genes. (DOCX 16 kb) [file 12575_2019_108_MOESM4_ESM.docx]

**Table S2 The list of female-biased expressed genes**

| **Female Biased** | | | | |
| --- | --- | --- | --- | --- |
| *A1bg* | *Cnr1* | *Gng13* | *Myo5b* | *Slitrk1* |
| *Abca4* | *Cntn4* | *Gpr155* | *Ndst4* | *Sorbs2* |
| *Ablim3* | *Cntn5* | *Gpr64* | *Neu3* | *Sp5* |
| *Adamts19* | *Cntnap5a* | *Grm4* | *Nkd1* | *Srcrb4d* |
| *Agpat9* | *Col12a1* | *Gxylt2* | *Nmnat2* | *Sulf1* |
| *Agtr1a* | *Colec12* | *Hist3h2ba* | *Nmur2* | *Sv2b* |
| *Akr1c14* | *Cpa2* | *Hmgcll1* | *Nos1* | *Tlr5* |
| *Akr1c18* | *Cpxm2* | *Hmgcs2* | *Npnt* | *Tmem132c* |
| *Amph* | *Crip3* | *Hpca* | *Nr2e3* | *Tmem171* |
| *Ano1* | *Csgalnact1* | *Igfbp2* | *Olfm1* | *Tmem174* |
| *Anpep* | *Cyp2f2* | *Il13ra2* | *Opn1sw* | *Tnfrsf19* |
| *Aox3* | *Cyp4f15* | *Irx3* | *Oprd1* | *Tns4* |
| *Apold1* | *Dclk1* | *Irx3os* | *P2rx6* | *Trhde* |
| *Arhgap29* | *Dio3os* | *Isx* | *Pde10a* | *Tshr* |
| *Arl5c* | *Dpp10* | *Itpr2* | *Pla2r1* | *Tsix* |
| *Atp6ap1l* | *Emx2* | *Kcnb2* | *Pld5* | *Wnt2b* |
| *Axin2* | *Emx2os* | *Kcnd2* | *Podxl* | *Wnt4* |
| *B3gat1* | *Epn3* | *Kcnh3* | *Ppp1r3g* | *Wnt9a* |
| *BC068157* | *Ereg* | *Kcnq1* | *Ppp2r2b* | *1190002N15Rik* |
| *Bcat1* | *Fam196b* | *Krt18* | *Prkg2* | *1700026D08Rik* |
| *Bmp2* | *Fam83g* | *Krt20* | *Ptger3* | *1810041L15Rik* |
| *Bmpr1b* | *Fam84b* | *Laptm4b* | *Rag1* | *2900005J15Rik* |
| *Cacna1d* | *Fat4* | *Lbx2* | *Rasa4* | *4930485B16Rik* |
| *Cacna1i* | *Fgfr2* | *Lgals3bp* | *Rasgrp1* | *4932418E24Rik* |
| *Calb1* | *Fmo1* | *Lgr6* | *Rec8* | *4932435O22Rik* |
| *Ccdc64* | *Foxl2* | *Lrrc3b* | *Rgs2* | *6330403A02Rik* |
| *Cd164l2* | *Foxl2os* | *Lrrc4* | *Rmst* | *LOC102632313* |
| *Cd44* | *Fst* | *Lrrn1* | *Rnf43* | *LOC102633615* |
| *Cdcp1* | *Gabra4* | *Lypd6* | *Rspo1* | *LOC102635621* |
| *Cdkn1b* | *Gabrb1* | *Lypd6b* | *Rspo2* | *LOC102636042* |
| *Cers4* | *Gas6* | *Lzts1* | *Runx1* | *LOC102636125* |
| *Ces1h* | *Gbp2* | *Mbnl3* | *Ryr2* | *LOC102636511* |
| *Ces2g* | *Gbp6* | *Mfi2* | *S100a1* | *LOC102638005* |
| *Chrm4* | *Gdpd2* | *Mir135a-2* | *Sez6l* | *D630039A03Rik* |
| *Cib2* | *Gm13003* | *Msh4* | *Shisa9* | *D830015G02Rik* |
| *Clec2l* | *Gm19217* | *Msx1* | *Slc5a4a* | *E330013P04Rik* |
| *Clstn2* | *Gm6705* | *Msx1os* | *Slc7a2* | *A730017C20Rik* |
| *Xist* | *Zbtb7c* |  |  |  |
