# Supplementary material for: Comprehensive Transcriptomic Analysis of Mouse Gonadal Development Involving Sexual Differentiation, Meiosis and Gametogenesis
Source: Biol Proced Online. 2019 Oct 15;21:20. doi: 10.1186/s12575-019-0108-y (PMC6794783; doi:10.1186/s12575-019-0108-y)
Supplement: Supplementary file 5 — Additional file 5: Table S3. A list of differential alternative splicing meiotic genes. (DOC 66 kb) [file 12575_2019_108_MOESM5_ESM.doc]

**Table S3** A list of differential alternative splicing meiotic genes

| **Gene Symbol** | **Exon Number** | **Splicing Type** | **Adjusted P** |
| --- | --- | --- | --- |
| *Cdk2* | 7 | Cassette | ＜0.05 |
| *Ddx4* | 11 | A3SS | ＜0.05 |
|  | 2 | AltStart | ＜0.05 |
| *Dmc1* | 6 8 11 | Cassette | ＜0.05 |
|  | 7 9 | Cassette_multi | ＜0.05 |
| *Dmrtc2* | 3 | AltStart | ＜0.05 |
| *Exd1* | 2 | Cassette | ＜0.05 |
| *Fancd2* | 39 | Cassette | ＜0.05 |
| *Hfm1* | 6 | MXE | ＜0.05 |
|  | 13 25 26 27 28 29 30 31 32 | Cassette | ＜0.05 |
|  | 26 | Cassette_multi | ＜0.05 |
|  | 39 | AltEnd | ＜0.05 |
| *Hormad1* | 15 | Cassette | ＜0.05 |
| *Hormad2* | 14 | Cassette | ＜0.05 |
| *Hsf1* | 10 | Cassette | ＜0.05 |
|  | 12 | Cassette | ＜0.05 |
| *Meiob* | 7 | Cassette | ＜0.05 |
|  | 15 | Cassette | ＜0.05 |
| *Msh4* | 16 | Cassette_multi | ＜0.05 |
| *Msh5* | 7 10 11 | Cassette | ＜0.05 |
|  | 6 | Cassette_multi | ＜0.05 |
| *Numa1* | 19 | Cassette | ＜0.05 |
|  | 4 | AltStart | ＜0.05 |
| *Piwil4* | 19 20 | Cassette | ＜0.05 |
|  | 12 | MXE | ＜0.05 |
|  | 8 | AltStart | ＜0.05 |
| *Prdm9* | 11 | IR | ＜0.05 |
| *Prkar1a* | 4 | AltStart | ＜0.05 |
| *Smc1b* | 2 3 4 5 6 7 18 | Cassette | ＜0.05 |
| *Spin1* | 7 | AltStart | ＜0.05 |
| *Spo11* | 5 | Cassette | ＜0.05 |
|  | 2 5 7 | AltStart | ＜0.05 |
|  | 12 | A5SS | ＜0.05 |
| *Stag3* | 19 | IR | ＜0.05 |
|  | 19 20 | Cassette_multi | ＜0.05 |
| *Sun1* | 12 | Cassette | ＜0.05 |
| *Syce2* | 3 | Cassette | ＜0.05 |
| *Syce3* | 2 | AltStart | ＜0.05 |
| *Sycp1* | 6 | Cassette | ＜0.05 |
| *Sycp2* | 8 | AltStart | ＜0.05 |
|  | 11 26 43 | Cassette | ＜0.05 |
| *Tdrd1* | 6 | Cassette | ＜0.05 |
| *Tdrd9* | 10 12 21 | Cassette | ＜0.05 |
|  | 24 | Cassette_multi | ＜0.05 |
|  | 35 | AltEnd | ＜0.05 |
| *Tex11* | 25 | AltEnd | ＜0.05 |
| *Tex14* | 35 | Cassette | ＜0.05 |
| *Tex15* | 3 | AltStart | ＜0.05 |
|  | 2 10 | Cassette | ＜0.05 |
| *Zfp318* | 11 | A3SS | ＜0.05 |
|  | 9 | Cassette_multi | ＜0.05 |
